# Parkinson’s disease in Gaucher disease patients: what’s changing in the counseling and management of patients and their relatives?

**DOI:** 10.1186/s13023-020-01529-y

**Published:** 2020-09-23

**Authors:** Maja Di Rocco, Alessio Di Fonzo, Antonio Barbato, Maria Domenica Cappellini, Francesca Carubbi, Fiorina Giona, Gaetano Giuffrida, Silvia Linari, Andrea Pession, Antonella Quarta, Maurizio Scarpa, Marco Spada, Pietro Strisciuglio, Generoso Andria

**Affiliations:** 1 Unit of Rare Diseases, Department of Pediatrics, IRCCS Istituto Giannina Gaslini, Via Gerolamo Gaslini 3, 16147 Genoa, Italy; 2 Neuroscience Section, Department of Pathophysiology and Transplantation, Dino Ferrari Center, IRCCS Foundation Ca’ Granda Ospedale Maggiore Policlinico, University of Milan Neurology Unit, Milan, Italy; 3 Department of Clinical Medicine and Surgery, “Federico II” University Hospital, Naples, Italy; 4 Department of Medical Science and Community, University of Milan, Milan, Italy; 5 Regional Referral Centre for Lysosomal Storage Diseases, Division of Internal Medicine and Metabolism, Civil Hospital, AOU of Modena, University of Modena and Reggio Emilia, Modena, Italy; 6 Department of Translational and Precision Medicine, Sapienza University, Rome, Italy; 7 Regional Reference Center for Rare Diseases, Clinical Division of Haematology and Transplantation, PO Ferrarotto Hospital, Azienda Ospedaliera-Universitaria Policlinico-Vittorio Emanuele, Catania, Italy; 8 Center for Bleeding Disorders and Coagulation, Careggi University Hospital, Florence, Italy; 9 Pediatric Unit, Department of Medical and Surgical Sciences, S. Orsola Hospital, University of Bologna, Bologna, Italy; 10 Center for Microcythemia, Iron Metabolism disorders, Gaucher disease-Hematology and Transplantation Unit, “A. Perrino” Hospital, Brindisi, Italy; 11 Regional Coordinating Center for Rare Disease, University Hospital of Udine, Udine, Italy; 12 Department of Pediatrics, AOU Città della Salute e della Scienza di Torino, University of Torino, Torino, Italy; 13 Department of Translational Medical Sciences, Section of Pediatrics, Federico II University, 80131 Naples, Italy; 14 Professor Emeritus “Federico II” University Hospital, Naples, Italy

**Keywords:** Gaucher disease, Risk of Parkinson’s disease, Counseling, Management

## Abstract

**Background:**

How to address the counseling of lifetime risk of developing Parkinson’s disease in patients with Gaucher disease and their family members carrying a single variant of the *GBA1* gene is not yet clearly defined. In addition, there is no set way of managing Gaucher disease patients, taking into account the possibility that they may show features of Parkinson’s disease.

**Methods:**

Starting from an overview on what has recently changed in our knowledge on this issue and grouping the experiences of healthcare providers of Gaucher disease patients, we outline a path of counseling and management of Parkinson’s disease risk in Gaucher disease patients and their relatives.

**Conclusion:**

The approach proposed here will help healthcare providers to communicate Parkinson’s disease risk to their patients and will reduce the possibility of patients receiving inaccurate information from inadequate sources. Furthermore, this resource will help to empower healthcare providers to identify early signs and/or symptoms of Parkinson’s disease and decide when to refer these patients to the neurologist for appropriate specific therapy and follow-up.

## Background

Gaucher disease (GD) is an inherited metabolic disorder caused by biallelic mutations in the *GBA1* gene. *GBA1* encodes the glucocerebrosidase (GCase) enzyme, which catalyses the hydrolysis of glucosylceramide into ceramide and glucose. Macrophages engorged with aberrant lysosomes, as a result of the GCase-impaired activity (Gaucher cells), infiltrate into the reticuloendothelial system of the affected organs [1].

GD type 1, which accounts for up to 95% of patients with GD in Europe and America, is typically considered a systemic disorder, without neurological involvement. Anaemia, leukopenia, thrombocytopenia with frequent bleeding, hepatosplenomegaly, osteopenia with bone pain, easy fractures, failure to grow and delayed puberty, bone marrow infiltration with bone medullary infarcts and osteonecrosis are the main features of this disease. Some GD patients with systemic disorder associated with neurological involvement are usually reported as affected with neuronopathic GD (GD type 2 and 3) [2].

The required criterion for the definition of neuronopathic GD is gaze palsy, predominantly horizontal, with slow or absent saccades. Some neuronopathic GD patients only show this sign, whilst others show different neurological abnormalities including cognitive impairment, ataxia, hyperreflexia, spasticity, cerebellar or action tremor, stridor, dysphagia, dysarthria, dystonia, seizures and progressive myoclonic epilepsy [3].

Age of onset at 6 months, rapid deterioration in the first 2 years of life and death by 4 years of age are characteristic of acute neuronopathic GD (GD type 2), whereas a later onset and neurological deterioration occurring only in subjects with the progressive myoclonic epilepsy variant are characteristic of chronic neuronopathic GD (GD type 3) [3].

Adult onset Parkinson’s disease (PD), multiple system atrophy and dementia with Lewy bodies are not features of neuronopathic GD [3]. However, it has been shown that the individuals with GD have an increased risk of developing PD compared to the general population [4, 5]. PD is a progressive neurodegenerative disorder characterised by several motor and non-motor signs and symptoms with a mean onset at 50–60 years of age. PD is primarily due to the degeneration of the midbrain dopaminergic neurons, however, in the later stage of the disease, the neuropathology may affect several different areas of the nervous system [6].

The diagnosis of PD is based on clinical criteria including rest tremor, rigidity, bradykinesia and a good response to levodopa intake. Other clinical features either motor (e.g., hypomimia, dysarthria, dysphagia, sialorrhea, shuffling gait, postural instability, festination, freezing, dystonia and micrographia), or non-motor (e.g., sleeping disorders, hyposmia, autonomic dysfunction and cognitive/behavioral abnormalities), may appear starting from several years before the diagnosis (prodromal phase) to later stages of the disease [7, 8]. Rapid eye movement sleep behavior disorder (RBD), a condition characterised by acting out of vivid, intense and violent dreams, is a prodromal marker of PD. [9]

The association between the presence of *GBA1* variants and an increased risk of developing PD was noticed in GD clinics more than 2 decades ago [10, 11]. In addition, the incidence of PD among GD patients and their relatives, who are carriers of the *GBA1* mutation, seem to be higher than in the general population [12, 13]. The important role of *GBA1* in the pathogenesis of PD was firmly established when larger populations of PD patients were screened worldwide [14]. Several studies confirmed the significantly higher incidence of *GBA1* mutations among PD patients compared to non-affected subjects in various populations [15].

*GBA1* mutations represent only a predisposing risk factor for PD. This implies that not every carrier will develop the disease. The reason for this reduced penetrance has not yet been fully elucidated. Some authors have tried to correlate the severity of the mutation with the age of onset and disease phenotype [16, 17]. However, the concept of severity of mutation is ambiguous with respect to GD, as mutation N370S (p.N409S) reported as a mild mutation could be related to severe systemic GD, while mutation L444P (p.L483P), reported as severe, is associated with a wide spectrum of clinical phenotypes, including mild GD. Furthermore, the concept of mutation severity is even more controversial in the field of PD risk because *GBA1* variants not associated with GD (e.g., E326K (p.E365K) and T369M (p.T408 M)), predispose to PD. [18] The reason of such variability is largely unknown.

Recent evidence suggests that other genetic factors, such as rare variants in other lysosomal genes, may play a role in increasing the susceptibility to develop PD in *GBA1* variant carriers, but a definitive role of the contribution of these variants requires further confirmation and is not yet usable for diagnostic purposes [19]. Moreover, whether PD phenotype in patients with GD differs from that in heterozygous *GBA1* carriers is still unclear. Nevertheless, it is well known that carriers of *GBA1* variants harbor an increased risk of developing PD that is 5 times higher in heterozygous carriers and 10–20 times higher in homozygous or compound heterozygous carriers, although, these risks vary in different populations [20, 21].

The issue on how to inform GD patients and their relatives has, so far, been overlooked for various reasons including the risk of PD not being high, there are no biomarkers to better assess the PD risk, there is no actionable content of the information, the communication could generate anxiety and stress and information on sensitive clinical conditions might spread and affect the subject in some fields as insurance, job and social life. However, something is changing.

A recent study surveyed subjects with GD for their knowledge of the increased risk of PD and interest in learning about it. Most GD patients asked to be informed about PD risk in the clinical setting by the physician in charge of GD patients at the time of GD diagnosis together with information concerning other comorbidities [21]. On the other hand, among PD patients, there is also a general lack of knowledge regarding the association between GD and PD risk [22].

In a survey involving adults who had *GBA1* screening and did not carry *GBA1* variants, the participants indicated with a high level of unanimity that they believe that healthcare providers should inform patients about the increased risk of PD prior to screening. They also expressed that learning this information would be important and beneficial and would not cause more anxiety than receiving news of being a *GBA1* carrier alone [23].

From another point of view, GD doctors might be sued for omitted, retarded, incompleted or potentially misleading information on PD risk to those who are potential candidates for future neuroprotective drugs or who already need a symptomatic treatment for early symptoms and signs of PD. Some longitudinal studies on *GBA1* variant carriers provide evidence of a progressive worsening of motor and non-motor prodromal PD features [24, 25]. The identification of *GBA1* variant carriers and the early identification of subjects, either with mono or biallelic *GBA1* mutations, who will develop PD is crucial to address patients to future neuroprotective drugs or at least to symptomatic treatments.

In this scenario, we propose a possible approach for the communication by health care providers to GD patients about the risk of PD and for management of this issue.

## Methods

The consensus working group was composed of 14 Italian specialists in internal medicine (*n* = 2), paediatrics (*n* = 5), hematology (*n* = 5), inborn errors of metabolism (*n* = 1) and neurology (*n* = 1), and are all actively involved in GD management and treatment.

For the first meeting all clinicians received a selected literature review about PD and GD. During the face-to-face meeting, all participants agreed that despite extensive literature on epidemiology of PD in subjects with GD and pathogenesis of PD in GD, scarce data apparently exists about counseling and clinical management of PD risk in GD patients. A neurologist (ADF) who follows most Italian patients with GD and PD, as well as subjects with PD heterozygous for *GBA1* variants, reported on his clinical experience and proposed which symptoms should be asked for early identification of PD. A pediatrician, also acting as chairman of an institutional ethics committee, advised on ethical issues concerning the communication of disease risk to asymptomatic subjects, including minors, and counseling of minors’ parents and caregivers.

The first meeting focused on the following topics:

- Communication of PD risk to adult patients with GD, to pediatric patients’ parents and caregivers and to subjects heterozygous for *GBA1* variants.
- Management model for early identification of PD by healthcare providers and referral to neurologists.

The second meeting was a conference call to reach consensus about some specific points of the two previously discussed issues. The first manuscript was revised by all participants and many suggestions were accepted after being collegially discussed. The final manuscript was approved by all participants.

### Communication of PD risk

In order to align practice with the patient’s preferences, it is important that GD patients receive the information on PD risk directly from their healthcare provider rather than being informed incidentally from websites, without any appropriate counseling [22]. The healthcare provider should counsel them, at diagnosis or as soon as possible during follow-up, about the increased risk of PD. The patient has the right to refuse to be informed on this risk. Patients must feel free to ask their physician for information and to have all doubts and possible misunderstandings clarified. They need to be reassured that the physician will eventually provide the necessary support for an early identification of PD signs and symptoms and, in case of symptoms affecting the quality of life, for an early therapeutic approach.

According to the current clinical practice, the physician should adapt the communication of the PD risk to the social and cultural background of GD patients or parents of underage GD patients. The communication should state clearly that the lifetime risk to develop PD affects both patients and heterozygous carriers, with the GD patients having a higher risk and an earlier onset compared to the heterozygous carriers. All GD patients who have been diagnosed before the emerging evidence of *GBA1* as a PD risk factor must receive this information as soon as possible.

Being the issue of the risk for PD among GD patients still an area of active investigation, the healthcare providers should keep uptodate on this issue.

**Fig. 1:**
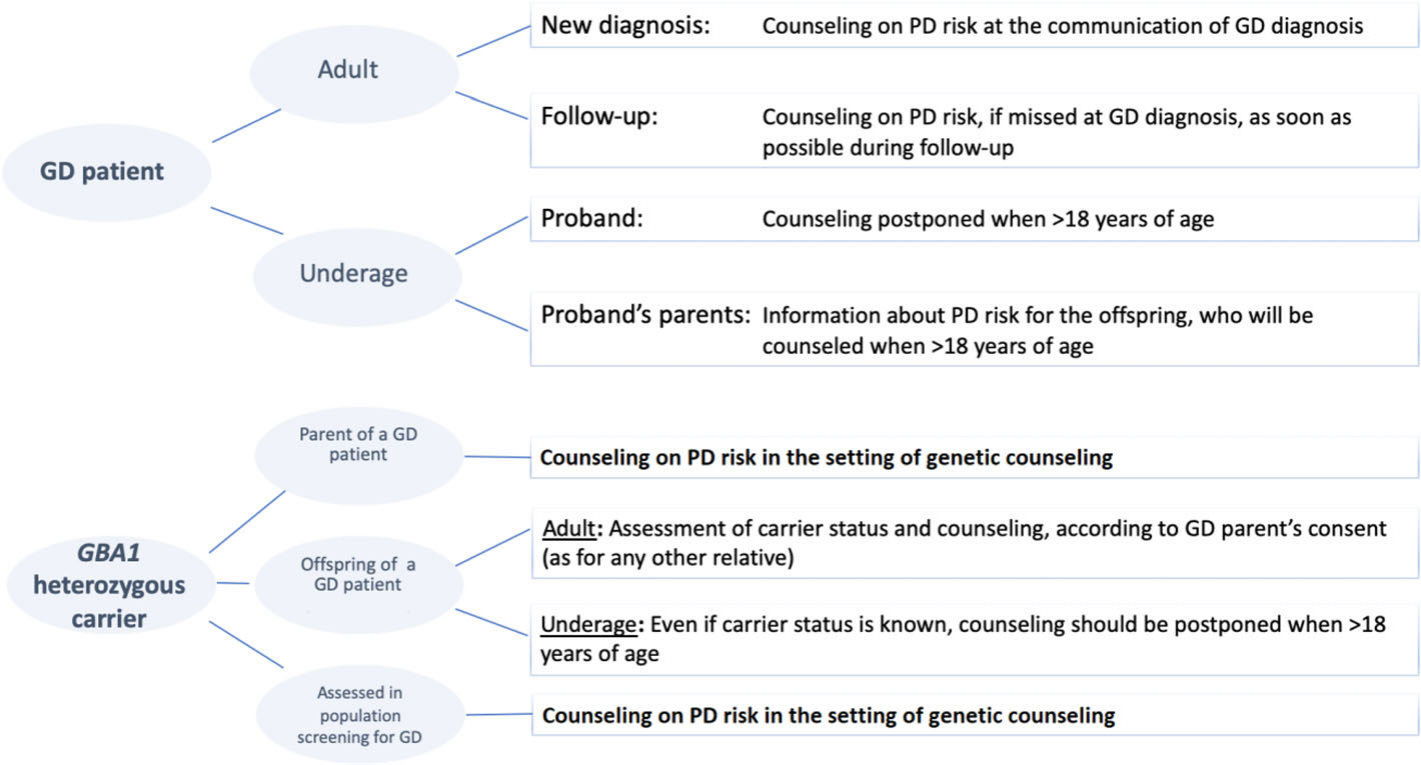
Communication of PD risk to GD patients and their relatives who are *GBA1* heterozygote mutation carriers

In case of GD diagnosis in an underage patient, parents should be informed at the time of the diagnosis about the increased risk of PD. However, since the PD onset rarely occurs before 40 years of age, it is inappropriate to inform the child. The information should be postponed to a later age, after transition from paediatric to an adult clinical setting and provided by the healthcare provider for adult patients. At the time of GD diagnosis in a young underage patient, if the status of heterozygous is confirmed in the parents, a genetic counselor should inform them of an increased PD risk (Fig. 1).

The panel reached consensus that the expanded familial screening to identify subjects carrying *GBA1* variants with an increased risk of PD is probably not justified because of the overall low risk of PD. However, patients or parents should be invited to decide whether to share with their relatives the information on the PD risk and the possibility for them to undergo genetic testing for *GBA1*, since this procedure does not seem to violate the principles of non-maleficence and justice. It is the duty of the physicians to offer information to the patients/parents to make a decision which respects their rights but also their relatives’ rights.

Genetic testing is offered frequently to couples of Ashkenazi Jewish ancestry, in the context of genetic counseling, since *GBA1* pathogenic variants are known to be frequent in that population. For these subjects it is appropriate to give the information about the increased PD risk, both for the GD offspring and for themselves, if they are GD heterozygotes.

### Management of PD risk

PD patients with GD or heterozygous for *GBA1* variants do not show specific features that would clearly distinguish them from patients with idiopathic PD. [17] However, the onset of PD is in average earlier in both heterozygous carriers and in subjects with GD, compared to the general population [12, 26].

**Fig. 2:**
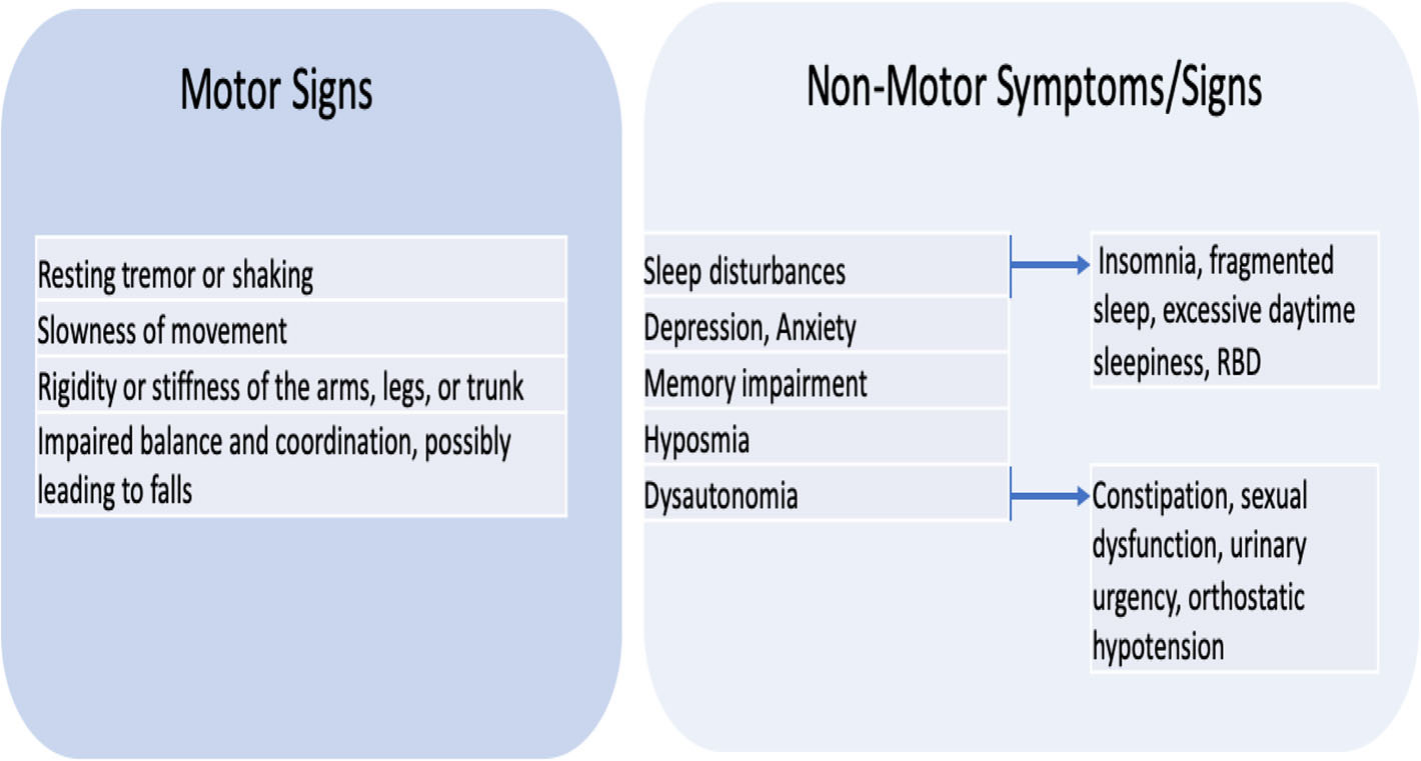
Early Motor Signs and Non-Motor Symptoms/Signs of PD

PD patients with GD or heterozygotes for a *GBA1* variant have a risk to develop dementia up to 3 times higher than patients with idiopathic PD. Hallucinations and RBDs are more common in *GBA1* PD patients, whereas there is no consensus yet on the frequency of other non-motor symptoms, such as anosmia, depression, anxiety, constipation, urinary symptoms, orthostatic hypotension and sexual dysfunctions. Increased incidence of dysautonomia and motor complications, such as dysphagia, dysarthria and freezing of gait, is reported in *GBA1* PD patients compared to idiopathic PD patients [12, 26]. Based on these data, the working group reached the following consensus concerning management of PD risk in GD patients:

- From the age of 35–40 years, every 12 months, healthcare providers should monitor the patient’s clinical history concerning non-motor and motor symptoms/signs suggestive of PD (Fig. 2) and to evaluate the patient for motor signs.
- The patient might be referred to neurologists when presenting with at least one clear motor sign alone or in the presence of any non-motor symptoms/signs with impact on the quality of life (Fig. 2).

## Conclusion

The risk for PD among GD carriers and affected individuals is still an area of active investigation.

However, according to current knowledge, we propose an evidence-informed practical guidance to communication, counseling and management of PD risk in GD patients. It is essential that healthcare providers with the updated evidence-based knowledge on this issue communicate the risk of developing PD to adult carriers of *GBA1* variants (either GD patients or their relatives who are heterozygous carriers), taking into account the cultural and emotional characteristics of each individual and their will to be informed.

Furthermore, this resource will help to empower healthcare providers to identify early signs and/or symptoms of PD and to decide when to refer these patients to the neurologist for appropriate specific therapy and follow-up.

A hopeful future and availability of neuroprotective therapies may influence the way in which we inform GD patients and *GBA1* variant carriers on their PD risk and will, therefore, require a new reflection on how to deal with the delicate issue of communicating the risk of this neurodegenerative disease.

## Data Availability

Not applicable.

## Abbreviations

GD: Gaucher disease
PD: Parkinson’s disease
GCase: Glucocerebrosidase
REM: Rapid eye movement
SBD: Sleep behavior disorder
